# The Course of COVID-19 and Long COVID: Identifying Risk Factors among Patients Suffering from the Disease before and during the Omicron-Dominant Period

**DOI:** 10.3390/pathogens13030267

**Published:** 2024-03-20

**Authors:** Mateusz Babicki, Damian Kołat, Żaneta Kałuzińska-Kołat, Joanna Kapusta, Agnieszka Mastalerz-Migas, Piotr Jankowski, Michał Chudzik

**Affiliations:** 1 Department of Family Medicine, Wroclaw Medical University, 51-141 Wroclaw, Poland; agnieszka.mastalerz-migas@umw.edu.pl; 2 Department of Biomedicine and Experimental Surgery, Medical University of Lodz, Narutowicza 60, 90-136 Lodz, Poland; damian.kolat@umed.lodz.pl (D.K.); zaneta.kaluzinska@umed.lodz.pl (Ż.K.-K.); 3 Department of Functional Genomics, Medical University of Lodz, Żeligowskiego 7/9, 90-752 Lodz, Poland; 4 Department of Internal Diseases, Rehabilitation and Physical Medicine, Medical University of Lodz, 90-647 Lodz, Poland; joanna.kapusta@umed.lodz.pl; 5 Department of Internal Medicine and Geriatric Cardiology, Medical Centre for Postgraduate Education, 01-813 Warsaw, Poland; piotrjankowski@interia.pl (P.J.); michalchudzik@wp.pl (M.C.); 6 Department of Nephrology, Hypertension and Family Medicine, Medical University of Lodz, 90-549 Lodz, Poland

**Keywords:** COVID-19, Omicron, pre-Omicron, long COVID, risk factors

## Abstract

SARS-CoV-2 has acquired many mutations that influence the severity of COVID-19’s course or the risk of developing long COVID. In 2022, the dominant SARS-CoV-2 variant was Omicron. This study aimed to compare the course of COVID-19 in the periods before and during the dominance of the Omicron variant. Risk factors for developing long COVID were also assessed. This study was based on stationary visits of patients after COVID-19 and follow-up assessments after 3 months. Clinical symptoms, comorbidities, and vaccination status were evaluated in 1967 patients. Of the analyzed group, 1308 patients (66.5%) were affected by COVID-19 in the period before the Omicron dominance. The prevalence of long COVID was significantly lower among patients of the Omicron group (47.7% vs. 66.9%, *p* < 0.001). The risk of long COVID was higher for women (OR: 1.61; 95% CI: 1.31, 1.99]) and asthmatics (OR: 1.46; 95% CI: 1.03, 2.07]). Conclusively, infection during the Omicron-dominant period was linked to a lower risk of developing long COVID. Females are at higher risk of developing long COVID independent of the pandemic period. Individuals affected by COVID-19 in the Omicron-dominant period experience a shorter duration of symptoms and reduced frequency of symptoms, except for coughing, which occurs more often.

## 1. Introduction

Coronavirus disease 2019 (COVID-19), caused by SARS-CoV-2, is an ongoing public health concern. Thus far, over seven million people have died from COVID-19, while the overall mortality rate is approximately 1–2% [1]. The virus was first identified in December 2019 in the Chinese city of Wuhan, but due to its rapid and uncontrolled worldwide spread, the World Health Organization (WHO) declared the COVID-19 pandemic in March 2020. Currently, most infected people have mild symptoms or are asymptomatic, but the virus also causes severe pneumonia, respiratory failure, and multiple organ dysfunction [2,3]. In addition to the risk of hospitalization and death due to COVID-19, some people have experienced symptoms that last for weeks or even months, which, depending on the duration of the symptoms, is defined as long COVID or post-COVID-19 syndrome [4,5,6]. These symptoms include fatigue, shortness of breath, cognitive and mental disorders, balance disorders, insomnia, smell/taste disorders, heart palpitations, chest pain, headaches, and musculoskeletal pain.

The course of COVID-19 depends on various factors, such as age, gender, obesity, immune response, and chronic diseases of the respiratory and cardiovascular systems. However, the clinical course of SARS-CoV-2 infection and the long-term effects of COVID-19 are also influenced by the evolution of the virus and the emergence of its new variants. Currently, the Centers for Disease Control and Prevention classify variants as those being monitored (VBMs), those of interest (VOIs), those of concern (VOCs), and those of high consequence (VOHCs) [7]. Over more than three years of the COVID-19 pandemic, five VOCs have been identified (i.e., Alpha, Beta, Gamma, Delta, and Omicron), which were responsible for subsequent waves [3,8,9].

Omicron is a SARS-CoV-2 variant that was classified by the World Health Organization as a VOC at the end of 2021 [3,10]. The emergence of this variant changed the trajectory of the COVID-19 pandemic. Despite its greater infectivity, caused primarily by its avoidance of the body’s humoral immunity acquired through vaccination or disease, Omicron causes a milder course of the disease and entails lower mortality compared to previous variants. These features allowed Omicron to quickly replace the earlier variant of SARS-CoV-2, i.e., the Delta variant [11,12,13]. Most cases of COVID-19 have been reported in Europe, where Omicron began to dominate in early 2022. The virus quickly spread to many regions and gradually evolved, giving rise to numerous subvariants. Initially, the BA.1 and BA.2 subvariants dominated, but over time, they were replaced by the BA.5, BA.2.75, BQ.1, and XBB.1.5 subvariants. All of these have shown the ability to avoid infection-induced and vaccine-induced neutralizing responses [3,13,14,15].

In many countries, healthcare systems were overwhelmed by rising infections during the Omicron wave of the pandemic. According to data provided by the WHO, Omicron is responsible for more than half of the 773 million confirmed cases of SARS-CoV-2 infection and has contributed to approximately one-fifth of the almost 7 million recorded deaths [1]. However, the actual number of infections and deaths associated with Omicron may be even higher. Possible reasons for this include the underdiagnosis of affected individuals and the failure to report new cases of the disease due to the WHO’s announcement that COVID-19 no longer constitutes a public health emergency of international concern [16].

Available reports have documented a milder COVID-19 course caused by the Omicron variant, lower frequency of hospitalizations and admissions to intensive care units, as well as a decrease in the mortality rate compared to previous pandemic waves [16,17,18]. However, there is a need for studies assessing the clinical diversity of cases and effects of SARS-CoV-2 during the dominance of Omicron, which is responsible for the longest wave of the pandemic. Therefore, our study aimed to analyze the course of COVID-19 and the long-term effects of the disease both before and during the dominance of the Omicron variant. Since SARS-CoV-2 continues to evolve and requires monitoring, such analysis is important for clinical evaluation of future SARS-CoV-2 variants.

## 2. Materials and Methods

**Figure 1:**
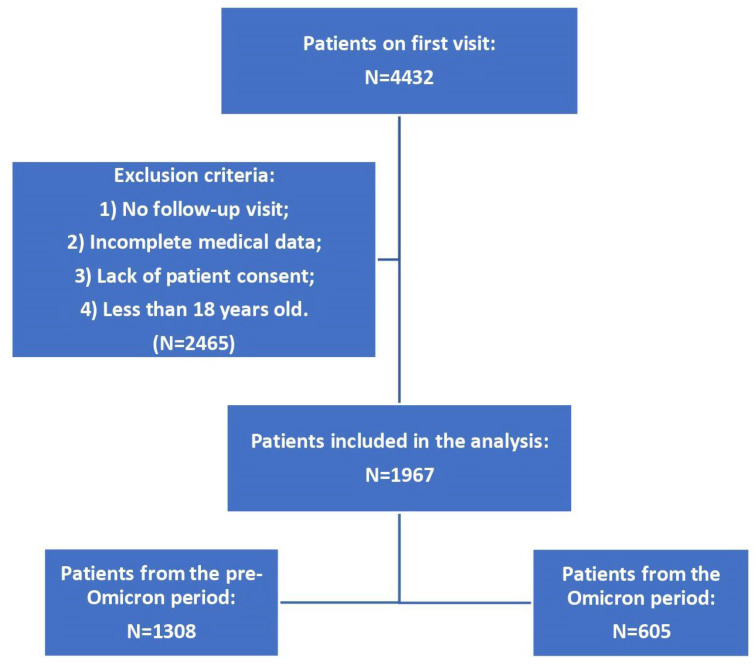
Flowchart of the study group.

This is a retrospective, longitudinal study analyzing data collected from direct patient visits to the medical doctor’s office as part of the STOP-COVID registry (ClinicalTrials.gov ID—NCT05018052). This registry includes Polish patients who had confirmed COVID-19 and went to see a doctor due to their ailments. As part of the program, patients underwent a follow-up visit after 3 months. Inclusion criteria for this study concerned those with a SARS-CoV-2 infection (confirmed via an antigen test or RT-PCR), aged ≥ 18 years, who gave written consent to participate in the study, and who had a follow-up visit 3 months after the end of their SARS-CoV-2 infection. Exclusion criteria concerned those with a lack of written consent, those aged < 18 years, those with no follow-up visit 3 months after their infection, and those with incomplete medical data (Figure 1).

Before participating in the study, patients were informed about the methodology and study goals, which allowed them to give their informed consent. The study was conducted per the guidelines of the Declaration of Helsinki and was approved by the Bioethics Committee of the Wroclaw Medical University (232/2022). During the first medical visit, the patients completed a health questionnaire that included sociodemographic data such as their age and gender. Information on their COVID-19 vaccination status and the occurrence of specific chronic diseases (such as diabetes, hypertension, asthma, hyperlipidemia, previous heart attack(s), coronary artery disease, and thyroid diseases) was also collected. Every patient who completed at least the basic vaccination regimen was considered a vaccinated person, regardless of the type of vaccine. The last stage of the questionnaire concerned clinical symptoms during COVID-19, their duration, and whether the disease required hospitalization. The analyzed clinical symptoms included temperature < 36.6 °C, temperature > 37.5 °C, coughing, shortness of breath (dyspnea), chest pain, smell and/or taste disorders, musculoskeletal pain, fatigue, headaches, diarrhea, vomiting, and hearing impairment. Subsequently, the patients were physically examined along with their anthropometric measurements (weight and height) being measured, on the basis of which their Body Mass Index (BMI) was calculated [19]. All of the above data enabled the division of patients into 5 groups, taking into account the severity of their COVID-19 [20]:

- Group 0 included people without clinical symptoms or with symptoms lasting up to 3 days;
- Group 1 consisted of patients treated at home with symptoms lasting up to 7 days;
- Group 2 comprised patients treated at home with symptoms lasting 7 to 14 days;
- Group 3 included patients treated at home with symptoms lasting at least 14 days and fever above 38 °C, shortness of breath, and oxygen saturation less than 94% for at least 3 days;
- Group 4 concerned hospitalized patients.

During the follow-up visit, patients completed a questionnaire assessing their persistent clinical symptoms. The following symptoms were evaluated: coughs, shortness of breath, fatigue, smell and/or taste disorders, hair loss, impaired concentration and memory (brain fog), chest pain, headaches, and musculoskeletal pain. According to the World Health Organization definition, long COVID was diagnosed in patients whose symptoms persisted for at least 3 months after SARS-CoV-2 infection [6].

For the purpose of this research, patients were divided into 2 groups on the basis of the infection period. Patients suffering from the disease between January 2020 and December 2021 were denoted as the “pre-Omicron” group. The second group consisted of patients suffering from the disease from January 2022 (when the dominance of the Omicron variant began in Poland [21]) to December 2022.

### Statistical Analyses

The analyzed variables were qualitative and quantitative. The normality of distribution was assessed using the Shapiro–Wilk test. A comparison of the qualitative variables was made using the chi-squared test, whereas the quantitative variables were analyzed using the non-parametric Mann–Whitney U test. The risk score for developing long COVID was calculated using a multivariate logistic regression model. Independent variables included age, gender, BMI, vaccination against COVID-19, disease period (pre-Omicron or Omicron), diabetes, hypertension, asthma, thyroid diseases, hyperlipidemia, previous heart attack(s), coronary heart disease, the presence of at least one chronic disease, and the severity of COVID-19. The dependent variable was the occurrence of long COVID. Three analogous models were made: the first was for the entire study group, the next one included patients suffering during the pre-Omicron period, and the last model concerned patients suffering during the dominance of the Omicron variant. Statistica 13.0 (StatSoft, Tulsa, OK, USA) was used for calculations. In all tests, the level of statistical significance was *p* < 0.05.

## 3. Results

### 3.1. Characteristics of the Study Group

The final analysis included 1967 patients who contracted COVID-19. Of the analyzed group of patients, 1308 (66.5%) were ill in the period before the Omicron dominance. Women encompassed a vast majority of the study group (65.7%). In total, 1591 (80.9%) patients were vaccinated against COVID-19, with the majority of vaccinations occurring during the pre-Omicron period (*p* < 0.001). The most common chronic diseases were hypertension and hyperlipidemia. There were no statistically significant differences in the occurrence of chronic diseases among patients of the pre-Omicron and Omicron groups. A detailed summary is presented in Table 1.

### 3.2. The Course of COVID-19

The analysis of the COVID-19 course showed that patients in the pre-Omicron period more often had a more severe infection and required hospitalization (*p* < 0.001). Moreover, patients’ symptoms persisted longer in the pre-Omicron period (12.4 ± 6.9 vs. 11.1 ± 7.5; *p* < 0.001). Both in the pre-Omicron period and during the Omicron dominance, three of the most common patient complaints included coughs, fatigue, and musculoskeletal pain. During the Omicron period, we observed a decrease in the number of patients with smell and/or taste disorders (21.3% vs. 55.2%; *p* < 0.001), temperatures < 36.6 °C (10.9% vs. 16.0%; *p* = 0.002), diarrhea (17.2% vs. 21.2%; *p* = 0.0034), fatigue (67.9% vs. 75.8%; *p* < 0.001), and headaches (55.1% vs. 60.4%; *p* = 0.031). On the other hand, patients of the Omicron group complained about coughing more frequently (76.6% vs. 65.1%; *p* < 0.001). In addition, a comparison of the pre-Omicron and Omicron periods only for patients vaccinated against COVID-19 showed convergent differences in the clinical picture of COVID-19. A detailed comparison of the vaccinated and unvaccinated patients is shown in Table S1, which is part of the Supplementary Materials. A detailed comparison of the clinical picture and course of COVID-19 is presented in Table 2.

### 3.3. Long COVID

The prevalence of long COVID was significantly lower among patients of the Omicron group (47.7% vs. 66.9%; *p* < 0.001). For both periods, the most common symptoms of long COVID were fatigue and memory/concentration problems. Among patients of the Omicron group, we noted a lower incidence of fatigue (25.8% vs. 38.1%; *p* < 0.001), shortness of breath (2.3% vs. 7.0%; *p* < 0.001), smell and/or taste disorders (2.3% vs. 4.8%; *p* = 0.006), and chest pain (2.4% vs. 6.0%; *p* < 0.001), as well as concentration and memory problems (7.0% vs. 13.1%; *p* < 0.001). Vaccinated patients also showed a higher incidence of long COVID, hair loss, fatigue, or smell and/or taste disorders. A detailed comparison of the long COVID pattern in the pre-Omicron and Omicron periods for the vaccinated and unvaccinated patients is shown in Table S2. A comparison of the long COVID clinical picture is presented in Table 3.

### 3.4. Risk Factors for Developing Long COVID

**Figure 2:**
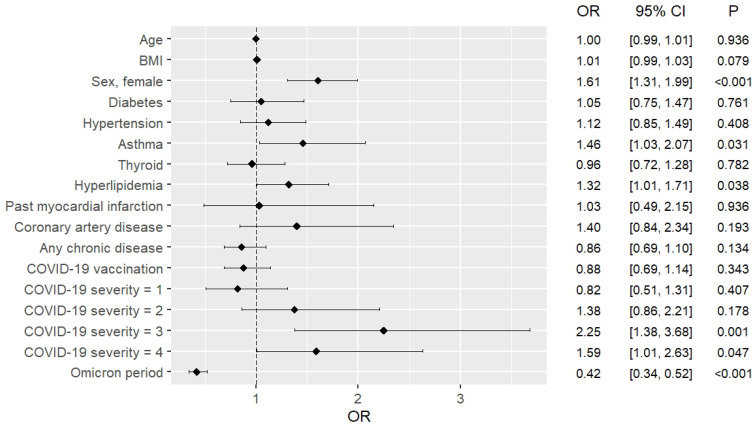
Complex logistic regression model assessment of the risk of developing long COVID in the whole group.

**Figure 3:**
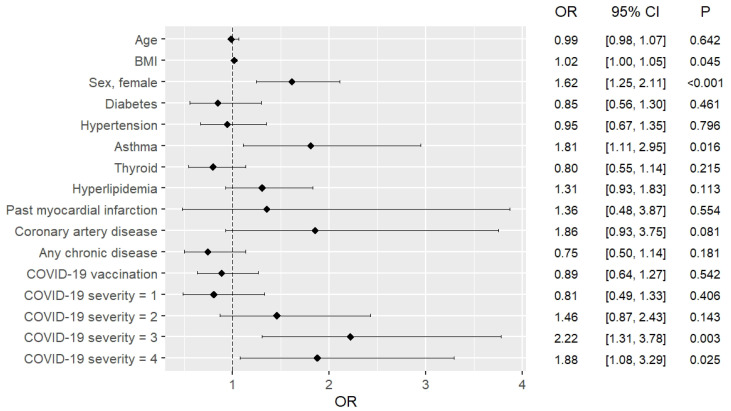
Complex logistic regression model assessment of the risk of developing long COVID in the pre-Omicron group.

**Figure 4:**
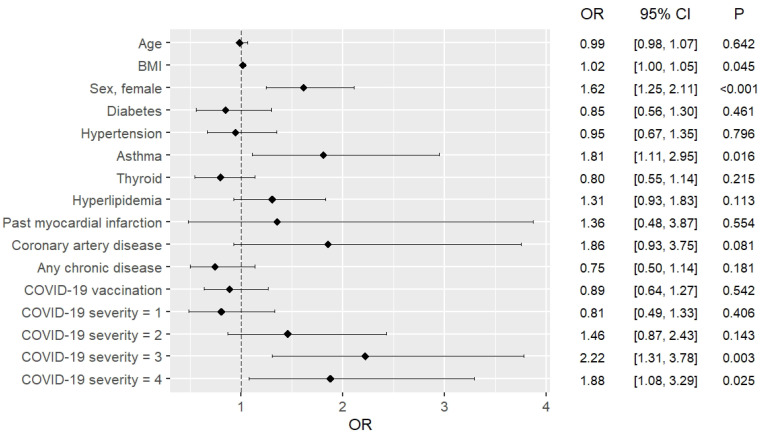
Complex logistic regression model assessment of the risk of developing long COVID in the Omicron group.

The complex logistic regression model showed that the risk of long COVID is higher for women (odds ratio [OR]: 1.61; 95% confidence interval [CI]: 1.31, 1.99]) and asthmatics (OR: 1.46; 95% CI: 1.03, 2.07]). Furthermore, this risk increases with the severity of COVID-19, but not among patients of the Omicron group. Nonetheless, it was also shown that infection during the Omicron-dominant period was associated with a lower risk of developing long COVID (OR: 0.42; 95% CI: 0.34, 0.52). The risk factors for the development of long COVID turned out to be similar among pre-Omicron patients and the entire study group regarding asthma, gender, and COVID-19 severity, although subtle differences were noted in terms of BMI and hyperlipidemia, with the former increasing the risk of long COVID in the pre-Omicron patients and the latter increasing the risk of long COVID in the entire cohort. Interestingly, only females had an increased risk of developing long COVID among patients of the Omicron group (OR: 1.74; 95% CI: 1.23, 2.53). A detailed summary of these results is presented in Table 4, as well as in Figure 2, Figure 3 and Figure 4.

## 4. Discussion

Following the identification of Omicron in November 2021 and its classification as a VOC shortly afterwards, this strain replaced Delta and became the dominant variant worldwide [3]. This variant also became dominant in Poland at the beginning of 2022 [21]. It was suggested that Omicron-infected individuals experience less severe symptoms than those infected with previous VOCs [22,23], which corresponds with the World Health Organization’s data indicating a lower severity of Omicron in comparison to Delta [24]. The increase in Omicron cases is caused by its high transmissibility, which complements its incubation time of around three days only [25,26]. Omicron bears more than sixty additional mutations relative to previous VOCs, including at least thirty mutations in the spike glycoprotein and fifteen mutations in the receptor-binding domain, causing quick attachment to human cells and immune evasion [27]. Neutralizing Omicron via the existing COVID-19 vaccination is less effective, thereby booster doses were advised to improve immunity and prevent hospitalization [28]. Furthermore, monovalent formulations targeting one of the Omicron subvariants, XBB1.5, have now been developed [29]. Although it is unclear whether we witnessed excessive mortality during the Omicron era (some studies suggest so [30], in opposition to data suggesting a lower contribution of Omicron to deaths compared to the Delta variant [31,32]), the absolute mortality remains high and healthcare systems are still overwhelmed by many patients who exhibit a spectrum of clinical presentations and outcomes [33]. Therefore, we aimed to assess the course of COVID-19 and long COVID, as well as to identify the risk factors of the latter among Polish individuals suffering from the disease before and during the dominance of the Omicron variant.

Our study indicates that pre-Omicron patients more often had severe infections, required hospitalization, and exhibited symptoms that lasted longer. During the Omicron period, a decrease in the incidence of smell and/or taste disorders, diarrhea, fatigue, headaches, and temperatures < 36.6 °C was observed. On the other hand, patients of the Omicron-dominant period were more likely to complain about coughing. A systematic review by Arabi et al. concluded that the Omicron variant is less severe, with a related pandemic wave reporting lower rates of hospitalization, intensive care unit admissions, deaths, and patients needing oxygenation/ventilation [34]. Other studies performed in Poland complemented these findings, indicating that the Delta variant is associated with a more severe disease course and a higher risk of death relative to Omicron [3,35]. Furthermore, the mortality of Omicron-infected individuals ranged from 0.01 to 13.1%, but those affected by previous variants ranked between 0.08% and 29.1%, with the proportions of intensive care unit admissions and mechanical ventilation being lower during the Omicron period [36]. However, it is important to note that some data report a comparable severity of Omicron infections relative to Delta [37], emphasizing that there is still a substantial risk for severe illness. As for the symptoms’ duration, the clinical trial by DeWitt et al. indicated a mean duration of 9.7 days in the pre-Delta period, while that of the Delta-dominant period was 8.4 days and that of the Omicron-dominant period was 5.9 days [38]. The ZOE COVID study noted that vaccinated patients infected with Omicron or Delta had symptoms for 6.87 days or 8.89 days, respectively. A similar result was found among those who had received two vaccine doses plus a booster, with 4.4 days for Omicron and 7.7 days for Delta [39]. The aforementioned clinical trial by DeWitt and colleagues also confirmed that some symptoms occurred less often during the Omicron period, including the loss of taste/smell, diarrhea, fatigue, headaches, and fever [38], reaffirming our findings. As for the more prevalent complaints of coughs during the Omicron-dominant period in our data, this conforms with the results of Vihta et al.’s study, in which Omicron infection was found to be less severe and presented with less taste/smell complications but more coughs and sore throats relative to COVID-19 caused by wild-type or previous variants [40]. Coughing was also the most common symptom in another Omicron subvariant-oriented study [41]. Kang et al. detailed clinical characteristics of persistent coughing after COVID-19 in the Omicron era using a pooled analysis of three different patient registries [42].

Moreover, we observed that among COVID-19 patients of the Omicron group, the prevalence of long COVID was significantly lower, and among the persistent clinical symptoms we noted a lower incidence of fatigue, shortness of breath, smell and/or taste disorders, chest pain, and concentration and memory problems. Females and asthmatics were found to be at higher risk of developing long COVID. The risk also increased with the severity of COVID-19, but this was no longer evident in the Omicron period, during which only the gender-related risk maintained the same trend as it showed in the pre-Omicron era. Antonelli et al. indicated that the incidence of long COVID was lower in the Omicron era [43], which corresponds to our findings and the research by Diexer et al., who concluded that Omicron infection is associated with a lower risk of developing post-COVID-19 syndrome [44]. Moreover, the latter authors evaluated persistent symptoms and indicated that after Omicron infection, individuals with the post-COVID-19 condition were less affected by smell and taste disorders in comparison to participants infected with the other variants. Indeed, the multicenter prospective cohort study by Gottlieb et al. confirmed that taste/smell disorders were the most evident differences between the Omicron and Delta periods, but other symptoms such as chills, feeling hot, fevers, and nausea also occurred less often in the Omicron era [45]. Our findings indicate less frequent concentration and memory problems, which were close to showing statistical significance (*p* = 0.077) in the Gottlieb et al. study with a trend towards a decreasing number of participants with prolonged symptoms (15.4% for the pre-Delta period, 12.1% for the Delta period, and 11.0% for the Omicron period). Regarding risk factors of long COVID, Bai et al.’s research confirmed that females are at higher risk of developing long COVID [46], which is in line with the multicenter study by Fernández-de-Las-Peñas and colleagues [47]. A recent systematic review of prospective cohort studies by Wolff et al. concluded that pre-existing asthma measured in a hospital-based population increases the risk of long COVID; however, the authors emphasized that this was assessed using low-certainty evidence related to population selection and exposure or outcome measurements [48].

The decreased severity of Omicron is thought to be due to a combination of decreased virulence, increased vaccination rate, and immunity from earlier infections [49]. Moreover, Omicron exhibits a reduced cleavage efficiency of the spike protein, which impairs entry mediated by transmembrane serine protease 2 [50]. Since most cells of the throat and nose lack this protein, Omicron invades them via endocytosis [51]. Consequently, despite the greater transmissibility of Omicron, it infects the upper respiratory tract, while other strains infect the lower respiratory tract, making Omicron less harmful to humans [34,52].

It is important to note that we did not find statistically significant differences related to COVID-19 vaccination, which contrasts with some data from the literature. A systematic review by Paul et al. indicated that primary vaccinations were short-lasting and less protective against Omicron relative to the Delta and Alpha variants. Although booster doses reestablished the vaccination’s effectiveness, they did so to a lower extent against Omicron [53]. Another comprehensive approach by Arabi et al. certified that earlier infection protects against Omicron, even though the degree of immunity was much lower relative to Delta. These authors concluded that optimal protection is not provided by vaccination or previous infection, but rather through hybrid immunity [54]. Lastly, Notarte et al. systematically reviewed the impact of COVID-19 vaccination on the risk of developing long COVID and related symptoms, revealing that the majority of analyzed papers reached a consensus regarding the improvement of long COVID symptoms following at least one dose post vaccination [55]. On the other hand, some studies did not find long COVID symptom alterations in the majority of patients [56,57,58]. Interestingly, the first mentioned study by Tsuchida et al. reported that patients with worsening long COVID symptoms had elevated antibody titer ratios due to excessive immune responses to vaccination [56]. Such discrepancies warrant further investigation of COVID-19 vaccine-related outcomes.

### Study Strengths and Limitations

Our study undoubtedly has several limitations. First, the registry includes patients who self-reported to a health center due to persistent ailments. Due to the above, some COVID-19 patients did not have a follow-up visit despite invitations. Moreover, the analyzed group is not representative of either the group of recovered patients in Poland or the Polish society; therefore, further observations on a representative group of patients are necessary. Another limitation is the lack of analysis of chronic diseases that may have had a significant impact on the disease course and complications, such as cancer or chronic kidney disease. It should also be noted that not all possible symptoms of long COVID were included in our analysis. Our database also lacks information on the pharmacotherapy used during SARS-CoV-2 infection. To assess the severity of the course, we used our division, which has already been used in previous research [20]. To the best of the authors’ knowledge, there are currently no validated tools to assess the severity of COVID-19 at home. It should also be mentioned that in Poland, there is no routine testing of all COVID-19 patients to assess the viral subtype, which may affect the final results. Nevertheless, during the analyzed periods of the Omicron subtype’s predominance, among the tests performed evaluating SARS-CoV-2 subtypes, more than 90% involved the Omicron variant [21]. The indisputable advantage of our study is the longitudinal nature of the observations and the personal visits of patients to the health center. Moreover, this study compares both the course of COVID-19 and the course of long COVID between patients suffering before and during the dominance of the Omicron variant.

## 5. Conclusions

The risk of developing long COVID increases with the severity of COVID-19, but not among patients suffering from the disease during the dominance of the Omicron variant. Nonetheless, infection during the Omicron-dominant period presents a lower risk of developing long COVID. Females are at higher risk of developing long COVID independent of the pandemic period. Individuals affected by COVID-19 in the Omicron-dominant period experience a shorter duration of symptoms and reduced frequency of symptoms (smell and/or taste disorders, diarrhea, fatigue, headaches, temperatures < 36.6 °C, shortness of breath, chest pain, concentration and memory problems), except for coughing, which occurs more often.

## Figures and Tables

**Table 1:** Characteristics of the study group, including the division of the group into the periods before and during the dominance of the Omicron variant.

Variable		Pre-Omicron (N = 1308)	Omicron (N = 659)	*p*-Value	The Whole Group (N = 1967)
Age		54.8 ± 13.2	55.7 ± 14.0	0.272 *	55.1 ± 13.5
BMI		27.9 ± 5.4	27.8 ± 5.9	0.153 *	27.8 ± 5.5
Gender:	Female	834 (63.8)	458 (69.5)	**0.011 #**	1292 (65.7)
	Male	474 (36.2)	201 (30.5)		675 (34.3)
Diabetes		136 (10.4)	72 (10.9)	0.719 #	208 (10.6)
Hypertension		509 (38.9)	263 (39.9)	0.669 #	772 (39.2)
Asthma		122 (9.3)	80 (12.1)	0.053 #	202 (10.3)
Thyroid diseases		236 (18.0)	112 (17.0)	0.566 #	348 (17.7)
Hyperlipidemia		277 (21.2)	133 (20.2)	0.608 #	410 (20.8)
Previous heart attack(s)		33 (2.5)	16 (2.4)	0.895 #	49 (2.5)
Coronary artery disease		78 (6.0)	37 (5.6)	0.755 #	115 (5.8)
Any chronic diseases		805 (61.5)	413 (62.7)	0.627 #	1218 (61.9)
COVID-19 vaccination		1114 (85.2)	477 (72.4)	**<0.001 #**	1591 (80.9)

N—number; *—Mann–Whitney U test; #—chi-squared test. Data presented as N (%) or mean ± standard deviation. Significant effects (<0.05) are marked in bold.

**Table 2:** Comparison of the clinical picture and severity of COVID-19 in the period before and during the dominance of the Omicron variant.

Variable		Pre-Omicron (N = 1308)	Omicron (N = 659)	*p*-Value	The Whole Group (N = 1967)
Temperature < 36.6 °C		209 (16.0)	72 (10.9)	**0.002 #**	281 (14.3)
Temperature > 37.5 °C		705 (53.9)	338 (51.3)	0.273 #	1043 (53.0)
Cough		851 (65.1)	504 (76.6)	**<0.001 #**	1355 (68.9)
Dyspnea		663 (50.7)	329 (49.9)	0.749 #	992 (50.4)
Chest pain		604 (46.2)	284 (43.2)	0.204 #	888 (45.1)
Fatigue		992 (75.8)	446 (67.9)	**<0.001 #**	1438 (73.1)
Musculoskeletal pain		921 (70.4)	465 (70.6)	0.945 #	1386 (70.5)
Smell and/or taste disorders		680 (52.0)	141 (21.3)	**<0.001 #**	711 (36.1)
Headaches		790 (60.4)	364 (55.1)	**0.031 #**	1154 (58.7)
Diarrhea		277 (21.2)	113 (17.2)	**0.034 #**	390 (19.8)
Vomiting		101 (7.7)	43 (6.5)	0.336 #	144 (7.3)
Hearing impairment		130 (9.9)	77 (11.7)	0.233 #	207 (10.5)
Duration of symptoms		12.4 ± 6.9	11.1 ± 7.5	<0.001 *	11.98 ± 7.1
COVID-19 course severity:	0	80 (6.2)	8 (1.2)	**<0.001 #**	88 (4.5)
	1	360 (27.5)	215 (32.6)		575 (29.2)
	2	364 (27.8)	228 (34.6)		592 (30.1)
	3	296 (22.6)	121 (18.4)		417 (21.2)
	4	208 (15.9)	87 (13.2)		295 (15.0)

N—number; *—Mann–Whitney U Test; #—chi-squared test; 0—patients without clinical symptoms or with symptoms lasting up to 3 days; 1—patients treated at home with symptoms lasting up to 7 days; 2—patients treated at home with symptoms lasting from 7 to 14 days; 3—patients treated at home with symptoms lasting at least 14 days, and with a fever greater than 38 °C, dyspnea, and oxygen saturation < 94% for at least 3 days; 4—hospitalized patients. Data presented as N (%) or mean ± standard deviation. Significant effects (<0.05) are marked in bold.

**Table 3:** Comparison of persistent clinical symptoms before and during the dominance of the Omicron variant.

Variable	Pre-Omicron (N = 1308)	Omicron (N = 659)	*p*-Value	The Whole Group (N = 1967)
Long COVID	875 (66.9)	314 (47.7)	**<0.001**	1189 (60.4)
Fatigue	498 (38.1)	170 (25.8)	**<0.001**	668 (33.9)
Cough	38 (2.9)	10 (1.5)	0.060	48 (2.4)
Dyspnea	92 (7.0)	15 (2.3)	**<0.001**	107 (5.4)
Smell and/or taste disorders	63 (4.8)	15 (2.3)	**0.006**	78 (4.0)
Musculoskeletal pain	55 (4.2)	17 (2.6)	0.062	72 (3.7)
Chest pain	79 (6.0)	16 (2.4)	**<0.001**	95 (4.8)
Hair loss	64 (4.9)	22 (3.3)	0.112	86 (4.4)
Concentration and memory problems	171 (13.1)	46 (7.0)	**<0.001**	217 (11.0)
Headaches	25 (1.9)	8 (1.2)	0.257	33 (1.7)

N—number. Data presented as N (%). Significant effects (<0.05) are marked in bold.

**Table 4:** Complex logistic regression model assessment of the risk of developing long COVID for the entire group and patients suffering from the disease before and during the dominance of the Omicron variant.

Variable		The Whole Group (N = 1967)		Pre-Omicron (N = 1308)		Omicron (N = 659)	
		OR [95% CI]	*p*-Value	OR [95% CI]	*p*-Value	OR [95% CI]	*p*-Value
Age		1.00 [0.99, 1.01]	0.936	0.99 [0.98, 1.07]	0.642	1,00 [0.99, 1.02]	0.561
BMI		1.01 [0.99, 1.03]	0.079	1.02 [1.00, 1.05]	**0.045**	1.01 [0.98, 1.03]	0.613
Gender, female		1.61 [1.31, 1.99]	**<0.001**	1.62 [1.25, 2.11]	**<0.001**	1.74 [1.23, 2.53]	**0.003**
Diabetes		1.05 [0.75, 1.47]	0.761	0.85 [0.56, 1.30]	0.461	1.53 [0.89, 2.67]	0.127
Hypertension		1.12 [0.85, 1.49]	0.408	0.95 [0.67, 1.35]	0.796	1.08 [0.98, 1.75]	0.098
Asthma		1.46 [1.03, 2.07]	**0.031**	1.81 [1.11, 2.95]	**0.016**	1.21 [0.71, 2.07]	0.489
Thyroid diseases		0.96 [0.72, 1.28]	0.782	0.80 [0.55, 1.14]	0.215	1.40 [0.87, 2.29]	0.168
Hyperlipidemia		1.32 [1.01, 1.71]	**0.038**	1.31 [0.93, 1.83]	0.113	1.26 [0.81, 1.96]	0.305
Previous heart attack(s)		1.03 [0.49, 2.15]	0.936	1.36 [0.48, 3.87]	0.554	0.71 [0.21, 2.45]	0.583
Coronary artery disease		1.40 [0.84, 2.34]	0.193	1.86 [0.93, 3.75]	0.081	0.80 [0.35, 1.83]	0.601
Any chronic diseases		0.86 [0.69, 1.10]	0.134	0.75 [0.50, 1.14]	0.181	0.69 [0.40, 1.10]	0.098
COVID-19 vaccination		0.88 [0.69, 1.14]	0.343	0.89 [0.64, 1.27]	0.542	0.83 [0.57, 1.19]	0.302
COVID-19 course severity:	0	Reference	Reference	Reference	Reference	Reference	Reference
	1	0.82 [0.51, 1.31]	0.407	0.81 [0.49, 1.33]	0.406	0.54 [0.13, 2.31]	0.409
	2	1.38 [0.86, 2.21]	0.178	1.46 [0.87, 2.43]	0.143	0.82 [0.19, 3.51]	0.794
	3	2.25 [1.38, 3.68]	**0.001**	2.22 [1.31, 3.78]	**0.003**	1.54 [0.36, 6.79]	0.559
	4	1.59 [1.01, 2.63]	**0.047**	1.88 [1.08, 3.29]	**0.025**	0.74 [0.17, 3.31]	0.693
Omicron period		0.42 [0.34, 0.52]	**<0.001**	---	---	---	---

N—number; OR—odds ratio; 95% CI—95% confidence interval; 0—patients without clinical symptoms or with symptoms lasting up to 3 days; 1—patients treated at home with symptoms lasting up to 7 days; 2—patients treated at home with symptoms lasting from 7 to 14 days; 3—patients treated at home with symptoms lasting at least 14 days, and with a fever greater than 38°C, dyspnea, and oxygen saturation < 94% for at least 3 days; 4—hospitalized patients. Data presented as OR [95% CI]. Significant effects (<0.05) are marked in bold.

## Data Availability

The data presented in this study are available on request from the corresponding author.

## Supplementary Materials

The following supporting information can be downloaded at: https://www.mdpi.com/article/10.3390/pathogens13030267/s1, Table S1: Comparison of the clinical picture of COVID-19 in the pre-omicron and omicron periods, with a breakdown of vaccinated and unvaccinated patients against COVID; Table S2: Comparison of the clinical picture of long COVID in the pre-omicron and omicron periods, with a breakdown of vaccinated and unvaccinated patients against COVID.
