# Supplementary material for: The Course of COVID-19 and Long COVID: Identifying Risk Factors among Patients Suffering from the Disease before and during the Omicron-Dominant Period
Source: Pathogens. 2024 Mar 20;13(3):267. doi: 10.3390/pathogens13030267 (PMC10975347; doi:10.3390/pathogens13030267)
Supplement: Supplementary file 1 [file pathogens-13-00267-s001.zip › pathogens-2866197-supplementary.pdf]

Table S1. Comparison of the clinical picture of COVID-19 in the pre-omicron and omicron periods, with a breakdown of vaccinated and unvaccinated patients against COVID-

		Vaccinated				Unvaccinated			
**Variable**		**pre-Omicron (N=1114)**	**Omicron (N=476)**	**p-value**	**The whole group (N=1590)**	**pre-Omicron (N=194)**	**Omicron (N=182)**	**p-value**	**The whole group (N=376)**
**Temperature <36.6°C**		177 (15.9)	61 (12.8)	0.112#	238 (14.9)	32 (16.5)	11 (6.0)	**0.001**#	43 (11.4)
**Temperature >37.5°C**		590 (53.0)	231 (48.4)	0.097#	821 (51.6)	115 (59.3)	107 (58.8)	0.923#	222 (59.0)
**Cough**		711 (63.8)	375 (78.8)	**<0.001**#	1086 (68.3)	140 (72.2)	129 (70.9)	0.782#	269 (71.5)
**Dyspnea**		569 (51.1)	232 (48.6)	0.372#	801 (50.3)	94 (48.5)	97 (53.3)	0.348#	191 (50.8)
**Chest pain**		510 (45.8)	196 (41.1)	0.084#	706 (44.4)	94 (48.5)	88 (48.6)	0.974#	182 (48.4)
**Fatigue**		843 (75.7)	325 (68.1)	**<0.001**#	1168 (73.4)	149 (76.8)	121 (66.5)	**0.025**#	270 (71.8)
**Musculoskeletal pain**		776 (69.7)	344 (72.1)	0.325#	1120 (70.4)	145 (74.7)	121 (66.5)	0.078#	266 (70.7)
**Smell and/or taste disorders**		588 (52.8)	23 (4.8)	**<0.001**#	611 (38.4)	92 (47.4)	8 (4.4)	**<0.001**#	100 (26.6)
**Headache**		654 (58.7)	272 (57.0)	0.263#	926 (58.2)	136 (70.1)	91 (50.0)	**<0.001**#	227 (60.4)
**Diarrhea**		233 (20.9)	73 (15.3)	**0.009**#	306 (19.2)	44 (22.7)	40 (21.9)	0.870#	84 (22.3)
**Vomiting**		87 (7.8)	29 (6.1)	0.223#	116 (7.3)	14 (7.2)	14 (7.7)	0.869#	28 (7.4)
**Hearing impairment**		108 (9.7)	56 (11.7)	0.218#	164 (10.3)	22 (11.3)	21 (11.5)	0.951#	43 (11.4)
**Duration of symptoms**		12.3 ± 6.8	11.0 ± 7.0	<0.001*	11.9 ± 6.9	13.0 ± 6.9	11.4 ± 8.6	**<0.001***	12.3 ± 7.8
**COVID-19 course severity**	0	69 (6.2)	4 (0.8)		73 (4.6)	11 (5.7)	4 (2.2)		15 (4.0)
	1	314 (28.2)	159 (33.4)		473 (29.8)	46 (23.7)	56 (30.8)		102 (27.1)
	2	303 (27.2)	176 (36.9)	**<0.001**#	479 (30.1)	61 (31.4)	52 (28.6)	0.141#	113 (30.1)
	3	248 (22.2)	85 (17.8)		333 (20.9)	48 (24.7)	36 (19.8)		84 (22.3)
	4	180 (16.2)	53 (11.1)		233 (14.6)	28 (14.4)	34 (18.7)		62 (16.5)

N – number; * – Mann U Whitney Test; # – Chi-square test; 0 – patients without clinical symptoms or with symptoms lasting up to 3 days; 1 – patients treated at home with symptoms lasting up to 7 days; 2 – patients treated at home with symptoms lasting from 7 to 14 days; 3 – patients treated at home with symptoms lasting at least 14 days and fever greater than 38°C, dyspnea, and saturation <94% for at least 3 days; 4 – hospitalized patients. Data presented as N (%) or mean ± standard deviation.

Table S2. Comparison of the clinical picture of long COVID in the pre-omicron and omicron periods, with a breakdown of vaccinated and unvaccinated patients against COVID-19.

**Variable**	Vaccinated				Unvaccinated			
	**pre-Omicron (N=1114)**	**Omicron (N=476)**	**p-value**	**The whole group (N=1590)**	**pre-Omicron (N=194)**	**Omicron (N=182)**	**p-value**	**The whole group (N=376)**
**Long COVID**	739 (66.3)	223 (46.8)	**<0.001**#	962 (60.5)	136 (70.1)	91 (50.0)	**<0.001**#	227 (60.4)
**Fatigue**	421 (37.8)	128 (26.8)	**<0.001**#	549 (34.5)	77 (39.7)	42 (23.1)	**0.005**#	119 (31.6)
**Cough**	33 (2.9)	8 (1.7)	0.138#	41 (2.6)	5 (2.6)	2 (1.1)	0.289#	7 (1.9)
**Dyspnea**	79 (7.1)	12 (2.5)	**0.003**#	91 (5.7)	13 (6.7)	3 (1.7)	**0.015**#	16 (4.3)
**Smell and/or taste disorders**	55 (4.9)	11 (2.3)	**0.016**#	66 (4.1)	8 (4.1)	4 (2.2)	0.288#	12 (3.2)
**Musculoskeletal pain**	48 (4.3)	12 (2.5)	0.085#	60 (3.8)	7 (3.6)	5 (2.8)	0.635#	12 (3.2)
**Chest pain**	67 (6.0)	10 (2.1)	**<0.001**#	77 (4.8)	12 (6.2)	6 (3.3)	0.189#	18 (4.8)
**Hair loss**	51 (4.6)	9 (1.9)	**0.009**#	60 (3.8)	13 (6.7)	13 (7.2)	0.858#	26 (6.9)
**Concentration and memory problems**	145 (13.0)	37 (7.8)	**0.002**#	182 (11.4)	26 (13.4)	9 (5.0)	**0.004**#	35 (9.3)
**Headache**	18 (1.6)	7 (1.5)	0.831#	25 (1.6)	7 (3.6)	1 (0.6)	0.083#	8 (2.1)

N – number. Data presented as N (%), # – Chi-square test;
